# Recent Advancements in Regenerative Approaches for Thymus Rejuvenation

**DOI:** 10.1002/advs.202100543

**Published:** 2021-05-07

**Authors:** Himal Sharma, Lorenzo Moroni

**Affiliations:** ^1^ MERLN Institute for Technology‐Inspired Regenerative Medicine Department of Complex Tissue Regeneration Maastricht University Maastricht 6229 ER Netherlands

**Keywords:** biofabrication, immune reconstitution, organoids, stem cells, T cells, thymus regeneration

## Abstract

The thymus plays a key role in adaptive immunity by generating a diverse population of T cells that defend the body against pathogens. Various factors from disease and toxic insults contribute to the degeneration of the thymus resulting in a fewer output of T cells. Consequently, the body is prone to a wide host of diseases and infections. In this review, first, the relevance of the thymus is discussed, followed by thymic embryological organogenesis and anatomy as well as the development and functionality of T cells. Attempts to regenerate the thymus include in vitro methods, such as forming thymic organoids aided by biofabrication techniques that are transplantable. Ex vivo methods that have shown promise in enhancing thymic regeneration are also discussed. Current regenerative technologies have not yet matched the complexity and functionality of the thymus. Therefore, emerging techniques that have shown promise and the challenges that lie ahead are explored.

## Graphical abstract

T cells defend the body against pathogens. They are developed in a factory, which is called the thymus. During very early in life the thymus starts to shrink leading to higher risks of infections. In this review, ways that can regenerate the thymus are discussed.

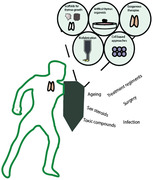

## 1 Introduction

The thymus is a primary lymphoid organ, which is located in the superior mediastinum above the heart.^[^ ^1^ ^]^ Its main function is for the maturation of an immunocompetent T cell repertoire. T cells have essential roles as mediators and effectors in the immune system. Defects in the thymus can lead to poor development of T cells, leading to ineffectiveness in combating various diseases.^[^ ^2^ ^]^

The thymus fully develops before birth, and after one year postnatal the number of thymic epithelial cells (TECs) begins to decrease in a process called thymic involution, where large parts of the gland populated by thymocytes are replaced with adipose tissue. The thymus loses cellularity and organization as part of the ageing process at a rate faster than other tissues, and cannot be reversed.^[^ ^3^ ^]^ A proposed mechanism for thymic involution in humans is puberty wherein there is an increased production of sexual hormones. This accelerates the decrease of thymocytes at a rate of 3–5% per year.^[^ ^4^ ^]^ With increasing age, there is a loss of naïve T cells and an increase in memory T cells. There is also a decrease in the diversity of the T cell repertoire. Consequentially, these changes result in a higher risk of contracting infections and failure to remove self‐reactive T cells.^[^ ^3^ ^]^

Moreover, T cells are observed to have functional defects in their activity with ageing. Studies have shown that there is a decline in expression of the markers CD3, CD28, and CD27 in aged mice, which can lead to impairments in their stimulation. Also, the JAK‐STAT pathway, which is important in signal transduction for T cell cytokine production and proliferation, is subdued.^[^ ^5^ ^]^

The importance of the thymus is further underscored as its absence or impairment causes severe diseases associated with immunodeficiency and autoimmunity. In genetic diseases like DiGeorge syndrome, a deletion on chromosome 22 results in the thymus failing to develop. Patients, in essence, have no adaptive immune system, which leads to opportunistic infections through various pathogens.^[^ ^6^, ^7^ ^]^

An altered thymic architecture is associated with various disease states. Thymomas, for example, are tumors derived from the epithelial cells of the thymus. The majority of these epithelial cells fail to express AIRE, which helps express tissue‐specific antigens (TSA's), needed for the selection of working T cells and removal of self‐reactive ones. This can contribute to conditions such as autoimmune diseases like myasthenia gravis, which is seen in about 20% of the patients.^[^ ^8^ ^]^ Patients often have autoantibodies against interleukins and interferons thereby inhibiting the polarization of naive T cells. Further impairment includes the failure to produce Foxp3 T regulatory cells.

Structural defects, such as thymic atrophy, result in the release of autoreactive T cells which are capable of infiltrating non‐lymphoid tissues and are associated with increases in TNF*α* and IL‐6 production, causing systemic inflammation.^[^ ^5^, ^6^, ^9^ ^]^

In addition to various disease states causing thymic dysfunction, the thymus is very sensitive to damage, which can come in the form of viral and bacterial infections, such as bacterial lipopolysaccharide and HIV.^[^ ^10^, ^11^ ^]^ Conditioning regimes for therapies, such as bone marrow transplant, as well as radiotherapy and chemotherapy can damage the thymus.^[^ ^12^ ^]^ Other factors, such as environmental stressors (i.e., glucocorticoids, hormones, inflammatory cytokines, and immunosuppressive agents), can reduce cellularity and thymocyte numbers.^[^ ^13^, ^14^ ^]^ The thymus is often removed (thymectomy) for better access to the heart in new‐born children, leading them to be susceptible to various infections.^[^ ^14^ ^]^

Due to the great importance of the thymus, certain therapeutics have been proposed to restore a dysfunctional thymus. Compounds such as growth hormones, insulin growth factor (IGF), numerous interleukins, and keratinocyte growth factor (KGF) have been reported to increase thymopoiesis, replenish hemopoietic stem cells, and provide protection against damage. However, these therapies are systemic, and it is unknown whether improvements are due to direct or secondary actions. Effects can be limited and transient, while also having unwanted side effects.^[^ ^3^ ^]^

Transplantation is a viable option for DiGeorge syndrome and athymic patients. It has been shown to be well tolerated and has a survival rate of ≈75%. However, as of February 2021 in the USA alone, there have not been enough donors for the demand of 107 000 patients on the waiting list of vital organs. Some studies have shown that transplantation patients with DiGeorge syndrome can lead to complications. For instance, 20% of patients receiving thymus transplants developed autoimmunity post‐transplantation; possibly due to challenges in getting neo‐vasculature to newly implanted thymic tissue.^[^ ^15^ ^]^

Despite being exquisitely sensitive to damage, the young thymus has a tremendous capacity for repair.^[^ ^16^ ^]^ On the other hand, the capacity for thymus and immune regeneration in the aged and elderly diminishes. Therefore, new regenerative medicine approaches and targeted therapies for the thymus would be desirable to ameliorate all the aforementioned issues.

In this review, our current understanding of the embryonic development of the components of the thymus and its microenvironment will be discussed first. Then, the development and functionality of T cells with a focus on signaling in development will be reviewed. Finally, we will explore regenerative engineering approaches of the thymus, the benefits they could have, their applications and the challenges we face.

## 2 Anatomy and Microenvironment

**Figure 1:**
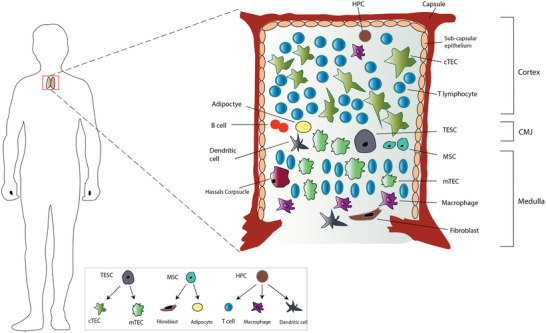
Diagram illustrating the anatomy of the thymus with its cellular constituents and the origin for each type of cell. The thymus is laid out in a 3D configuration, which enables T cells to develop through interactions with various cells. cTECs and mTECs are derived from thymic epithelial stem cells in the thymus (TESC). Fibroblasts and adipocytes are derived from mesenchymal stem cells (MSC), while T cells and myeloid cells are derived from hematopoietic cells (HPC) from the bone marrow.

The thymus is an organ composed of two lobes, which are separated by septa. Septa are made of connective tissue and reticular fibers. Each lobule is made of thymic epithelial cells (TECs) interspersed with various other cell types, which form an outer tightly packed cortex, and an inner, less dense medulla. TECs are organized in spongy 3D networks interspersed with many cell types including endothelial cells, dendritic cells (DCs), adipocytes, fibroblasts, macrophages, and B cells. The area between the cortex and medulla, the cortico‐medullary junction (CMJ), is where lymphoid precursors enter the thymus via blood vessels from the bone marrow. The CMJ is enriched with a mixture of mature and immature T lymphocytes as well as DC and B cells, which contribute to self‐tolerance. The number of B cells increases with age^[^ ^17^ ^]^ (**Figure** **1**).

Thymocyte precursors interact with the TECs to commit to the T cell lineage, and the release to the periphery is under thymic control. Epithelial cells are divided into distinct populations based on their antigen expression and structural characteristics, and are grouped into four epithelial subtypes: subcapsular cortical, inner cortical, medullary, and Hassall's corpuscles (HC).^[^ ^18^ ^]^

The cortical epithelial cells (cTECs) express Notch receptor ligands DLL1 and DLL4, which are important for precursor cells to commit to the T cell lineage. cTECs mediate the expansion of T cells via various growth factors and mediate positive selection. MHC associated peptides are formed by the proteasome subunit *β*5T exclusively expressed on cTECs. *β*5T (encoded by Psmb11) is a catalytic subunit, which mediates the presentation of ligands for MHC1 molecules, essential for the selection of competent T cells.^[^ ^19^, ^20^, ^21^ ^]^ cTECs are characterized by the keratin markers cytokeratin C8 and 18 (K8, K18). cTECs possess long cytoplasmic processes, which allow interactions with other cells. They can combine to form complexes, which are called thymic nurse cells (TNCs). TNCs are only detected postnatally and not in embryonic development. Previous research has suggested TNCs to be indispensable for T cell selection processes. However, Nakagawa et al. found TNCs to represent a subpopulation of cTECs that express *β*5T thymoproteasomes and suggested they can enwrap thymocytes. TNCs were shown to provide a microenvironment that can optimize T cell selection through secondary TCRa rearrangement in CD4 and CD8 T lymphocytes, but is not necessary for T cell development.^[^ ^22^ ^]^ Large numbers of developing lymphocytes are also present, but have a short life. Macrophages, which phagocyte them, also populate the thymus.^[^ ^23^ ^]^

The medullary epithelial cells (mTEC) are oval shaped, have short cytoplasmic extension and microvilli. mTECs are characterized by the keratin markers cytokeratin 4 and 5 (K4, K5). In addition, in the medullary area, there are a large amount of HC, macrophages, and DCs.^[^ ^24^ ^]^ mTECs express chemokines CC19 and CCL21, which home positively selected T cells and mediate negative selection.^[^ ^25^ ^]^ As a result, T cells are more mature and have larger cytoplasms than the cortical T cells. mTECs also express the transcription factor AIRE, which is essential for the negative selection of developing T cells. Studies have shown that AIRE can promote the expression of many TSAs on mTECs.^[^ ^26^ ^]^ There is now evidence that the display of AIRE also affects the removal of autoreactive T cells and the selection of Tregs.^[^ ^27^, ^28^ ^]^ Interestingly, another transcription factor, namely Forebrain‐expressed zinc finger 2 (Fezf2), was shown to be critical for the regulation of tissue restricted antigen (TRA) expression, independent of AIRE. Fezf2 target genes also differed from AIRE. Mice lacking Fezf2 in mTECs developed autoantibodies and inflammatory cell infiltration in peripheral organs.^[^ ^29^ ^]^

Discrete cTEC and mTEC zones are critical for thymic function. However, these groups are heterogeneous with many subpopulations with unique structural, molecular, and functional features.^[^ ^30^, ^31^, ^32^ ^]^ The mechanisms that govern adult TECs, that is, distinct cTEC and mTEC zones and their respective origins, are not fully understood. Previous lineage tracing studies described single cells, which could give rise to both cTECs and mTECs in the post‐natal thymus.^[^ ^33^, ^34^ ^]^ However, their molecular properties were poorly defined, and it was unclear whether these cells possessed the capacity to self‐renew—a hallmark of stem cells. Recent studies have identified progenitor cells with the expression of markers, such as CD205, *β*5t ,and IL7YFP.^[^ ^35^, ^36^ ^]^

Wong et al. defined a rare subset of TECs (CD45−EpCAM1+) capable of self‐renewal, which yielded both cTECs and mTECs.^[^ ^37^ ^]^ TEC subsets expressing PLET1(+)Ly‐51(+), which were likewise able to generate both cTECs and mTECs, have also been identified, suggesting the presence of a bipotent progenitor cell.^[^ ^38^ ^]^

Progenitor cells commit to the cTEC lineage by default. However, commitment to the mTEC lineage requires activation of the transcription factor NFkB.^[^ ^39^ ^]^ On the other hand, unipotent progenitors for cTECs and mTECs have also been described. For instance, mTECs expressing claudin‐3 and 4 can give rise to AIRE^+^ specific mTECs. While progenitors that express the markers EpCAM^+^ CD205^+^ and CD40^−^ give rise to cTECs.^[^ ^40^ ^]^ A deeper understanding is needed to know to what extent these progenitor cells play a role in postnatal TEC maintenance and regeneration, and their precise phenotypic markers.

Vasculature of the thymus holds great importance, as defects can cause poor architecture, as well as incorrect gene expression. The thymus has somewhat of a blood thymus barrier, similar to the blood‐brain and blood‐testis barrier.^[^ ^41^ ^]^ The lumen of the blood vessels is separated from the thymic parenchyma by endothelial cells, collagen, epithelial cells, and a perivascular space amongst other components. This barrier's function is to stop circulating antigens to come into contact with developing T cells. The barrier shows heterogenicity within the thymus. It is most prominent in the cortical area where there are heavy restrictions to the developing thymocytes. Blood vessels here are characterized by impermeable endothelial junctions and are rarely fenestrated. However, in the medullary area, the barrier is structurally incomplete. Vessels consist of arterioles and postcapillary venules. These leaky vessels have few endothelial junctions allowing antigens access into the thymic parenchyma.^[^ ^42^ ^]^

However, there is some dispute to the existence and role about the blood thymus barrier. Studies have shown that various pathogens can pass through the barrier and productively infect the thymus, leading to damage and consequently a higher susceptibility to infection. Interestingly, the permeability of the thymus can be influenced during pregnancy by molecules, such as steroids ^[^ ^42^ ^]^

DCs in the thymus are subdivided into two subsets: classical conventional DC (cDC) and the plasmacytoid lineage (pDC). They play an important role in negative selection in the medulla, as developing T cells with high affinity to self‐peptides are either deleted or differentiate into T regulatory cells. B cells are also present in the thymus, but only constitute 0.3% of the population. They are able to present antigens and express costimulatory molecules, which enable interaction with T cells and can efficiently mediate T cell selection. Yamano et al. observed that B cells could display enhanced APC features, such as increased levels of MHCII and CD80 and upregulated AIRE. They highlighted the complex interplay between B cells and T cells, where B cells could harbor tolerogenic features onto T cells. The interplay of B cells and T cells was explored further by Huang et al. where they showed that only B cells, which had undergone class switching, could promote the negative selection of autoreactive T cells. Whereas, when B cell class switching was inhibited, T cells showed autoreactivity. However, it is unclear what is the relative contribution that B cells play in tolerance.^[^ ^43^, ^44^ ^]^

The HC are formed by epithelial reticular cells and grow larger with age. Their functional relationship with other cells in the thymus remains elusive. However, they are known to mediate the growth of DC via the cytokine TSLP and believed to play a role in cell destruction. Other cell types may also be present in low numbers in the thymus, such as neuroendocrine cells. However, their roles remain unknown.^[^ ^45^ ^]^

## 3 Embryological Development

Knowing development mechanisms at the base of a functional thymus is important as defects can lead to immunodeficiency and/or autoimmunity. The thymus and parathyroid gland are simultaneously derived from the pharyngeal region of the embryo. They start as two flask‐shaped tubes, which extend ventrally. The pharyngeal region is composed of bulges called pharyngeal arches, which are separated from each other by structures called pharyngeal clefts and pouches. The clefts are invaginations composed of epithelial cells, which separate the arches. The pouches are composed of endoderm, which has an outer layer of epithelium. The thymus is derived from the endoderm of the third pharyngeal pouch.^[^ ^46^ ^]^

At day E9, a wave of mesenchymal cells derived from the neural crest (NC) populates the pouch. The mesenchymal cells populating/interacting with the arches are very critical for thymus organogenesis. In experiments in birds, removal of the NC resulted in failure of thymic organogenesis. In addition, defects in genes expressed in the NC, that is, PAX3 or HOXA3, led to an impairment of the thymus. Mesenchymal cells also influence thymic development by supplying growth factors. Mesenchymal cells release growth factors FGF7 and FGF10, which bind to receptors FGFR2IIIb on TECs. Studies showed that deficiencies in receptors resulted in aberrant thymic epithelium growth, suggesting a critical role of growth factors supplied by the mesenchyme cells.^[^ ^47^ ^]^

At day E10, the pouches and mesenchymal cells proliferate to form the bilateral primordia, where the mesenchymal cells will eventually form the capsule of the thymus. This proliferation occurs until day E12.5. At this stage, clear distinctions between the thyroid and thymus are visible.^[^ ^48^ ^]^ They then migrate to their final anatomical locations. Between this process, lymphocyte progenitors migrate into the thymus from the bone marrow in very low numbers at E11. Migration of precursors is characterized by occurring at precise stages in waves and is not a continuous process. Larger waves of lymphocyte migration are observed between E12 and E14.^[^ ^49^ ^]^

After day E12.5, the epithelial cells within the thymic primordium proliferate and subsequently differentiate into cTECs and mTECs. Shortly after, at day E13.5 expression of genes like MHCII is detectable. On E15.5 and E17.5, CD4^+^ and CD8^+^ SP T cells thymocytes are present.^[^ ^50^, ^51^ ^]^

In the human thymus, the medulla develops from week 8 and clear distinctions between the cortex and medulla can be seen by week 16. At week 8, other cells begin to infiltrate the thymus, such as vascular and mesenchymal cells, while the HC are only formed between 6–10 months. By week E14‐16 naïve T cells start to leave the thymus and populate the peripheral immune system.

The cTECs and mTECs are derived from the endoderm.^[^ ^49^ ^]^ Gordon et al. showed that following isolation and transplantation of the pharyngeal endoderm a functional thymus can be formed, which contains both epithelial regions and is not dependent on the pharyngeal ectoderm, contrary to previous findings.^[^ ^52^ ^]^

Transcription factors and signaling pathways play a critical role in thymic embryogenesis. Forkhead family transcription factor (FOXN1) is the earliest marker, which is strongly expressed on E11 of gestation and has a paramount role in TEC proliferation and differentiation. It should be noted that FOXN1 does not establish thymus fate, as TEC differentiation consists of FOXN1 dependent and independent stages and its exact role is not clear. However, FOXN1 was shown to have a critical role in early thymus organogenesis, since the thymi of mice that lacked FOXN1 were deficient in attracting T cell progenitors and supporting T cell lineage commitment.^[^ ^53^ ^]^ FOXN1 targets several hundred genes, some of which control mechanisms such as antigen processing and presentation. A notable FOXN1 target is Psbm11, which encodes for the proteasomal component *β*5T.^[^ ^54^ ^]^ FOXN1 direct role in influence TEC associated genes was further explored by Tamaka et al. who identified a FOXN1‐binding cis‐regulatory element near the *β*5T coding sequence. This was validated in mice where this region was essential for FOXN1 mediated gene transcription to produce CD8 T cells. Other notable FOXN1 targets include CD83, a surface marker on cTECs needed for the development of CD4SP cells.^[^ ^55^ ^]^

Finally, signaling pathways such as Shh, BMP, Wnt, and FGF play a role in the development of the early thymus. BMP plays a role in the epithelial–mesenchymal interaction as previously described. FGF is essential for the proliferation of thymic epithelia. Systemic administration of FGF7 increased its ability to support the development of T cells while Shh directed the initial parathyroid fate. Wnt proteins have been reported to mediate the expression of FOXN1 by binding to Frizzled receptors on epithelial cells.^[^ ^56^ ^]^ However, signaling for the initiation of organogenesis and patterning is poorly understood and would provide better insights into thymic differentiation.

## 4 T Cell Development

The adult thymus is colonized by subpopulations of hematopoietic stem cells (HSC) that migrate to the thymus directly via blood vessels into the CMJ. Thereafter, precursors begin to commit toward the T cell lineage and are characterized by the expression of a T cell receptor (TCR). Commitment to the thymocyte lineage depends strongly on the Notch signaling pathway. cTECs express Delta‐like Notch 1 (DLL1) and Delta‐like Notch 4 (DLL4), which interact with T cell precursors to differentiate T cells at the expense of B cells. Thymocytes can be divided into four subsets in their development: double negative (DN) (CD4^−^ CD8^−^), double positive (DP) (CD4^+^CD8^+^), CD4 single positive (SP) (CD4^+^CD8^‐^), and CD8 SP (CD4^−^CD8^+^) T cells. Progenitors then migrate to the subcapsular region of the cortex by certain cues. Precursors expressing the receptors CCR7 and CCR9 are attracted by a gradient of several chemokines’ ligands CCL21, CCL25 CXCL12, which are expressed by TECs.^[^ ^57^ ^]^

DP cells must express a TCR, which is capable of engaging the complex MHCI or MHCII, which will yield CD8 and CD4 SP T cells respectively. This process is known as positive selection and takes place in the cortical regions whereby cells with suboptimal expression of TCR will die. SP cells then migrate to the medulla and will undergo negative selection, where cells that bind too strongly will be signaled to undergo apoptosis to prevent autoimmunity. Negative selection takes place in both DP and SP stages. APCs of hematopoietic origin, such as DCs and macrophages, contribute to around 50% of this process.

The ability of the adaptive immune system to respond to an unlimited number of antigens and pathogens is due to the process of VDJ recombination, where random DNA arrangements are made, which yield diverse TCRs. TCRs are created from a variable and a constant domain. The variable domain consists of two disulfide chain composed of either an alpha (*α*) and beta (*β*), or a gamma (*γ*) and delta (*δ*) chain. This region is responsible for recognizing peptides (*γδ* T cells) or antigens, which are presented on MHC complexes (*αβ* T cells) through a specific area called the complementarity determining region (CDR). These chains can be constructed by several variants of variable (V), diversity (D) and joining (J) gene segments. These segments stochastically rearrange in a complex process, so that the TCR is specific for a certain antigen.^[^ ^58^ ^]^

### 4.1 Notch Signaling in T Cell Development

**Figure 2:**
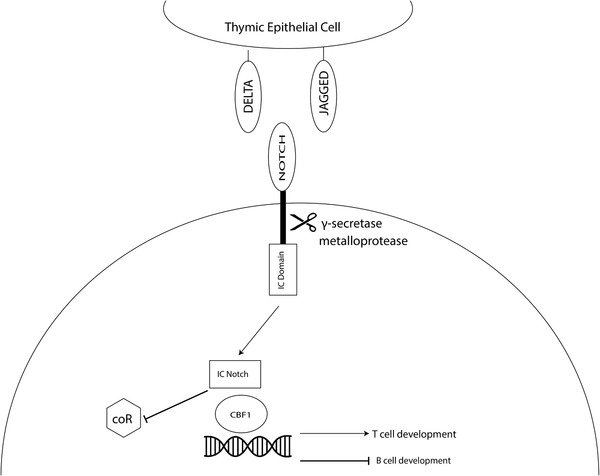
Notch signaling in T cell development. Notch receptors are expressed on precursor cells (bottom) interact with cTECs (above). Binding leads to the cleavage of the intracellular domain of Notch (IC domain), which can inhibit co‐repressors (coR) and activation of transcription factors (CBF1), which promote genes involved promoting cell differentiation/inhibition. Notch signaling is critical for T cell commitment.

Notch signaling influences T cell lineage commitment in the thymus. Notch is a transmembrane receptor expressed on thymocytes (mostly Notch1 and Notch3), which interacts with delta‐like (DLL1, DLL4) and Jagged ligands.^[^ ^59^ ^]^ These ligands are expressed on thymic stromal cells. This interaction leads to proteases, metalloprotease, and *γ*‐secretase to cleave the intracellular domain of Notch, releasing it to the nucleus. The intracellular domain of Notch activates the transcription factor CSL (CBF1), an activator of gene transcription for T cell development and removes transcriptional repressors. After the intracellular domain binds to the transcriptional factor CSL, this dislocates repressors Mint and NRarp and recruits activators like Mastermind (Maml), which enhances transcription of genes involved in promoting or inhibiting differentiation. Well known targets include HES1, which is a transcriptional repressor. However, its role in T cell development is not fully understood (**Figure** **2**). For instance, overexpression of HES1 does lead to an inhibition of B cell development. However, mice with a lack of HES1 have DN thymocytes present.^[^ ^60^ ^]^

The absence of Notch or DLL4 has shown to lead cells to differentiate into other lineages, such as B, NK, or myeloid cells. Studies have shown that the overexpression of Notch induces T cell differentiation. In addition, Notch signaling is known to preferentially lead to *αβ* over *γδ* T cells as well as helping the maturation from DN to DP. Finally, it can mediate *αβ* T cells to start expressing CD4 and CD8 co‐receptors.

### 4.2 Wnt Signaling in T Cell Development

**Figure 3:**
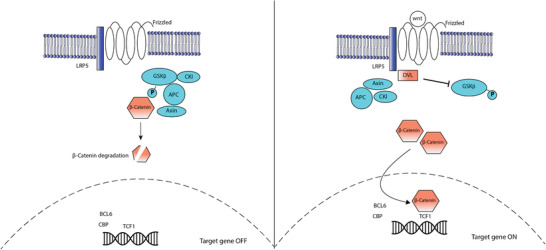
Wnt signaling in T cell development. Left: In an off‐stage, absence of Wnt results in *β*‐catenin being degraded by the destruction complex. Right: Wnt is observed to bind to frizzled, which dephosphorylates *β*‐catenin allowing it to translocate to the nucleus to bind to target genes Tcf1, which allows the development of T cells.

The Wnt family is known to have a role in T cell development, which has not been elucidated yet. Wnt proteins are produced by TECs and bind to Frizzled and LRP5 or LRP6 co‐receptors, which mediate the protein *β*‐catenin. Without Wnt signaling, *β*‐catenin is held in the “destruction complex”, phosphorylated and degraded. When Wnt is active, *β*‐catenin is no longer degraded and its dephosphorylation enables it to activate the Tcf/Lef transcription factors inside the nucleus, which allows the development of T cells (**Figure** **3**).^[^ ^61^, ^62^ ^]^

Studies have reported that Wnt supports the proliferation of DN thymocytes and is required for the transition of DN to DP, as shown by blocking Wnt binding to Frizzled, which inhibited T cell development at the DN stage.^[^ ^38^ ^]^ Wnt signaling is essential for the survival of CD4^+^ and CD8^+^ T cells. In addition, in a mouse model, the loss of Wnt signaling caused a loss of thymic architecture. Mice overexpressing Axin (a component of the destruction complex) showed fewer thymic cell numbers and increased apoptosis.^[^ ^62^ ^]^

### 4.3 Functionality of T Cells

After T cells have matured and undergone selection in the thymus, they migrate to the periphery. They comprise different subsets, which can respond to novel antigens, differentiate into memory T cells, as well as producing Tregs, which keep the immune system under control. CD4^+^ T cells are comprised of Th1, Th2, Th17, T follicular helper (Tfh), and Treg cells, which are characterized by cytokines and transcription factors that activate them and their effector functions.

Th1 cells are differentiated through the transcription factor T‐Bet. Th2 cells defend against parasites that are created through the transcription factor GATA3 under the influence of cytokines IL‐4 and IL‐5. Th17 cells enhance neutrophil response and release cytokines IL‐6 and IL‐21 and their differentiation is mediated through TGF‐ *β*. Tfh cells, on the other hand, activate B cells, and their differentiation from naïve T cells is mediated by IL‐6 and transcription factor BCL6 and T‐Bet. Finally, Tregs suppress other T cells to keep the immune response under control and limit the damage. They are characterized by the transcription factor FoxP3 and release anti‐inflammatory cytokines TGF‐*β* and IL‐10.^[^ ^63^ ^]^

Unlike CD4^+^ T cells, CD8^+^ T cells play an active role in directly killing foreign microbes through the release of perforin and granzyme causing apoptosis, leading to DNA damage. There are two major subsets of *γδ* T cells: (i) V9/V2T cells and (ii) a smaller subset of V1 and V3 *γδ* T cells. Unlike *αβ* T cells, *γδ* T cells do not need an MHC complex for antigen recognition. V2 *γδ* T cells recognize antigens in the form of alkylamines, bacterial phosphorus antigens, while V1 T cells are activated by alarm signals like heat shock proteins through MHC related molecules MICA/B.^[^ ^64^, ^65^ ^]^ V2 *γδ* T cells have anti‐tumour properties against disease like non‐Hodgkin B cell lymphoma, while V1 T cells have shown to infiltrate various cancers like colorectal, renal, and pancreatic ones. However, roles of *γδ* T cells can be dual as secreted cytokines can enhance tumor formation by increased angiogenesis via IL‐17 secretion and suppression of anti‐tumor cells.^[^ ^65^ ^]^

## 5 Regenerative Approaches of the Thymus

The regeneration of the thymus would offer a lot of therapeutic potential for patients and would improve their immune competence. This section will focus on the following: a) cell‐based approaches; b) organoid and scaffold‐based technologies; c) modulating endogenous repair pathways and exogenous administration of compounds for the regeneration of the thymus; and d) finally new biofabrication technologies, which could aid regenerative approaches.

### 5.1 Cell Based Approaches

Embryonic stem cells (ESCs) differentiate into many cell types, hold great promise for regenerative medicine, and are not as scarce as tissues and adult thymic stem cells. ESCs are derived from the inner cell mass of a blastocyst, which is an early stage in embryonic development at 4–5 days. ESCs can differentiate into all three primary germ layers, ectoderm, endoderm, and mesoderm.^[^ ^66^ ^]^

**Figure 4:**
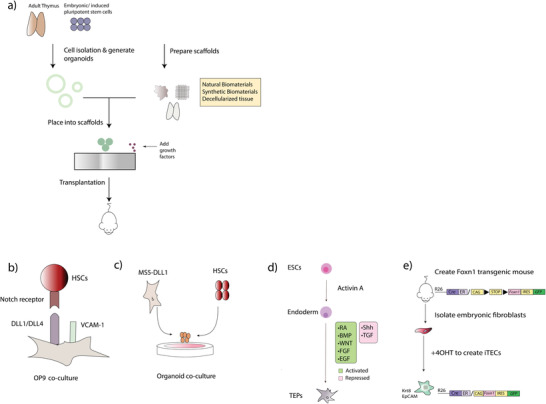
Regenerative approaches of the thymus. a) Illustration of the approaches to generate organoids using scaffolds. Briefly, TECs are extracted either from primary/embryonic tissue or created from ESCs/iPSC. Thereafter, they are expanded in scaffolds made from either natural polymer, synthetic materials, or decellularized tissues, which can be transplanted. b) OPDL1 culture system. OP9 cells are induced to express DLL1/4 (virally or synthetically), which differentiates HSCs into the T cell lineage. VCAM‐1 can be added to OP9 cultures, which acts synergistically with DLL4 to enhance T cell differentiation in a serum free culture. c) MS5‐DLL1 cells can be centrifuged and aggregated with HSCs to make organoids in a 3D co‐culture system. d) ESC‐derived TECs. ESCs are first subjected to Activin A to induce the formation of the definitive endoderm. Subsequently, various signaling pathways are modulated to develop TEPs. e) Reprogramming cells into TECs. Briefly, FOXN1 was knocked into a Rosa26 locus. Crossing them with Rosa26 CRERT2 mice yielded Rosa26CreERt2/CAG−STOP−FOXN1−IRES−GFP mice. Fibroblasts were isolated from embryos that were induced to overexpress FOXN1 upon the administration of tamoxifen (40HT), which reprogrammed them into TEC like cells with the expression of Krt8+ and EpCAM (termed iTECs). Other studies have augmented FOXN1 in mice as a treatment for a deteriorated thymus as seen in ref. [89].

Studies have demonstrated efficient commitment from human ESCs (hESCs) to the thymic precursor lineage through the regulation of a combination of different factors, which enables TEC modeling by altering their development process. Parent et al. generated human thymic epithelial precursors in vitro first by subjecting ESCs to Activin A, which led to the formation of the definitive endoderm. Subsequently, the signaling pathways TGF‐*β*, BMP4, retinoic acid, Wnt, Shh, and FGF were modulated, which generated thymic epithelial progenitors (TEPs).^[^ ^67^ ^]^ (**Figure** **4**d). TEPs were then transplanted into athymic mice, which yielded TECs that showed resemblance to endogenous tissue. Consistent with control models, the generated TECs expressed chemokines, such as CCL25, CC19, CCL21, as well as DLL4 on cTECs. As a result, the thymus was able to generate both CD4^+^ and CD8^+^ T cells, which were detected 10 weeks post transplantation in the peripheral blood. T cells also expressed the CD3 complex (a component of TCR), which showed diversity as analyzed through spectra typing. There were variations in the CDR3 length, shown by RT‐PCR, due to stochastic insertions/deletions created through VDJ recombination with variants in length forming a bell‐shaped curve (Gaussian distribution). Complexity can be measured by taking the average length of RT‐PCR products in addition to other methods.^[^ ^68^, ^69^ ^]^ Results from DNA spectral analysis of the CDR3 showed that V*β* regions had higher complexity scores than controls, suggesting proper gene rearrangement had ensued. This made it possible for T cells to develop into mature cells in a stepwise fashion (DN‐DP‐SP) and to be able to support both positive and negative selection. T cells were deemed to be functional as they could respond to TCR stimulation. For instance, cells could mount an immune response against implanted allogeneic skin grafts, indicating their capability in recognizing mismatches in MHC molecules. T cell proliferation was also observed, following anti‐CD3/CD28 stimulation, which correlated to reduced carboxyfluorescein succinimidyl ester (CSFE) levels.^[^ ^67^ ^]^

**Figure 5:**
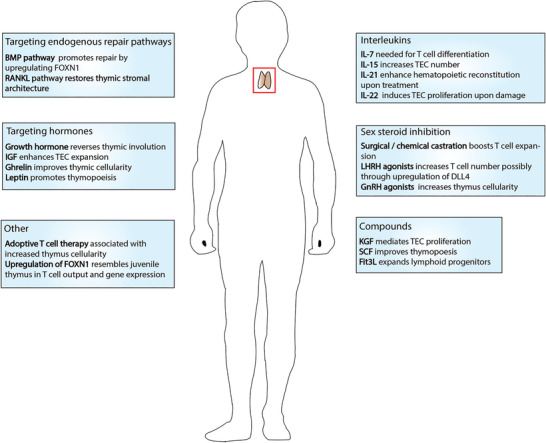
Illustration describing different strategies to enhance the thymus and improve T cell recovery.

In a similar study, Sun et al. used a similar method of generating TECs through the regulation of the retinoic acid, BMP, and Wnt signaling pathways in hESCs. Upon transplantation into humanized athymic mice, TECs were capable of producing operative T cells. T cells were able to proliferate to anti‐CD28 stimulation observed in a mixed leukocyte reaction assay. The T cells showed a response to PMA and concanavalin A (Con A) stimulation and were able to mature, yet to a limited extent. For instance, despite detecting a few DP T cells they did not observe any CD8^+^ T cells outside the thymus when organoids were cultured with HSCs.

Analyzed TEC grafts displayed thymic markers like MHCII. Unlike Parent et al., these TECs displayed AIRE, albeit its expression was detected throughout the tissue rather than showing expression restricted to the medullary area, which is what is normally seen in endogenous tissue. Discrepancies in these results could be in part due to the approaches in transplanting TECs. Sun et al. used human embryonic fibroblasts as a support system prior to transplant into humanized animal models. However, Parent et al. did not make use of such a support system and grafts were transplanted in nude mice.

Collectively, using human ESCs to generate TECs has still not matched the endogenous thymic integrity and T cell output. For instance, Parent et al. revealed there was an unusual cytokeratin expression, which indicated abnormal thymic architecture.^[^ ^67^, ^70^ ^]^ This could explain why these studies observed poor T cells number and functionality. For example, the number of CD4^+^ CD8^+^ T cell count was <20% compared to normal tissue.^[^ ^70^, ^71^ ^]^ Various other factors did not match the integrity of the normal thymus. For instance, the diversity of the CDR3 receptor scored significantly less in the spectral score analysis than the wild type (WT) model. Response to stimulation was not as efficient as endogenous tissue shown by thymic constructs only reaching 47% max CFSE production, while WT reached 87%. CD4^+^ T cells were also less efficient in rejecting allogenic skin grafts compared to controls, by taking much longer. This could be due to the downregulation of important genes for developing thymocytes such as FOXN1, DLL1, and CLC25 as opposed to the endogenous tissue.^[^ ^67^ ^]^

Consideration should be also given to the fact that the grafts were human derived and transplanted in murine models owing to a lack of xenogeneic‐graft interactions, leading to deficiencies in thymus and T cell function. Furthermore, despite the transcriptional control of early T cell development being well conserved, there are differences between mice and humans which could influence T cell development.^[^ ^72^ ^]^

To try to refine the differentiation of TECs from hESCs, Su and colleagues devised an improved protocol to efficiently yield functional TECs. The authors used a similar protocol to Parent et al. to generate TECs from ESCs; however, they added other compounds such as EGF, FGF7, FGF10, BMP4, FOXN1, and HOX3A. As a result, they observed high amounts of T cells in the periphery which produced cytokines IL‐2 and IFN‐ *γ* in response to anti‐CD3 stimulation. In addition, the survival time of T cell development was 24 weeks.^[^ ^73^ ^]^

Studies have shown the translational promise that cell‐based approaches may hold.^[^ ^74^ ^]^ Su et al. generated TECs from mouse embryonic stem cells (mESCs), which gave rise to functional Tregs. The importance of Tregs is highlighted in autoimmune disease, such as Type 1 diabetes (T1D) as mice with Treg defects have an accelerated onset of T1D. mESCs derived TEPCs were transplanted into a mouse model with T1D, which generated Tregs capable of suppressing the immune response. First, the number of Tregs in the primary and secondary lymphoid organs were 2–3 fold higher than controls. T effector cells were stimulated with anti‐CD3 in the presence of T regs and were measured by [^3^H] thymidine. Tregs from the transplanted organoids showed a twofold higher suppressive activity of T effector cells compared to controls (no organoids). Furthermore, mice with transplanted organoids did not develop T1D; however, 53% of the controls developed T1D.^[^ ^74^ ^]^ In addition, Tregs produced from mESC‐derived TECs have shown to be capable of preventing chronic graft versus host disease (cGVHD).^[^ ^75^ ^]^ Organoids productively formed working Tregs, which could accept allogenic grafts by suppressing CD4^+^ and CD8^+^ DP cells. TECs are a known target of cGVHD, and can impair negative selection and the generation of Tregs.^[^ ^76^, ^77^ ^]^ These organoids could be applicable for patients who have undergone bone marrow transplantation and often suffer from cGVHD. However, caution should be taken as there are risks of using allogeneic ESCs in addition to the possibilities of tumor formation. Interestingly, Lai et al. described that following implantation of ESCs derived TECs, cells did not lead to GVHD and enhanced graft versus tumor activity following bone marrow transplantation.^[^ ^78^ ^]^

Another challenge of transplanting organoid is that their survival time is relatively low. Due to the thymus’ bulky nature, the long term survival of grafts post transplantation of adult thymuses was poor.^[^ ^79^ ^]^ A study observed that the engraftment of TECs in the kidney capsule led to a lack of production of T cells and the degeneration of them in 2–4 weeks, which could be due to a lack of vascularity.^[^ ^80^ ^]^ To circumvent this, Chung et al. used lentiviral mediated expression of vascular endothelial growth factor in TECs, which enhanced the size of thymic implants and improved naïve T cell production up to tenfold.^[^ ^80^ ^]^ In addition, a higher expression of critical genes such as AIRE and DLL4, as well as a better organization of the thymus, were observed. Other methods to get around rejection have been described in cell‐based approaches, for instance, by creating TECs co‐expressing host and donor MHC class molecules, which could induce tolerance.^[^ ^81^ ^]^

Protocols using ESCs are complicated and extensive, and optimal conditions are yet to be defined. It remains to be seen whether these methods could be translated to humans. Nevertheless, these studies demonstrate that hESCs can support murine T cell production.

### 5.2 Induced Pluripotent Stem Cell (iPSC) Based Strategies for TEC Formation

Another possible solution to avoid the rejection of MHC mismatch in allogeneic recipients is through the use of iPSC TECs.^[^ ^82^, ^83^ ^]^ For instance, Chhatta et al. used autologous fibroblasts and reverted them to an iPSC stage. They were subsequently differentiated into TECs capable of producing T cells upon engraftment into athymic mice. Upon inspection of generated iPSC derived TECs, the authors measured a DLL4 expression twofold higher than controls and expressed immature TEC markers K5 and K8. However, other important markers like MHCII and Ly51^+^ for TEC morphology were not tested. Nevertheless, isolated T cells were functional, as they could produce IFN‐*γ* in response to anti‐CD3 stimulation.^[^ ^83^ ^]^ It would be interesting to see whether iPSC TECs are able to generate a broad repertoire of T cells from various sources of blood, especially naturally occurring lymphoid myeloid progenitors.

IPSCs unlike ESCs do not have ethical and regulatory constraints and theoretically would not encounter rejection due to originating from the host. However, problems like genomic instability and abnormal epigenetics can lead to immunogenicity.^[^ ^66^ ^]^

#### 5.2.1 Reprogramming Cells into TECs

An alternative to cell‐based approaches is reprogramming cells into TECs. Brendenkamp et al. created a transgenic mouse where embryonic fibroblasts were isolated and reprogrammed to TECs (termed iTECs) by tamoxifen activatable FOXN1 expression (Figure 4e). Prior to transplantation, iTECs were aggregated with fetal thymocytes and mesenchymal cells, which formed thymus like structures upon transplantation, which contained TEC subtypes.

This allowed the development of functional T cells that were remarkably comparable to a native thymus, as well as displaying a similar distribution and ratio of CD4^+^ to CD8^+^ T cells. Besides, there was a diverse repertoire of T cells; and the distribution of TCR*β* and TCR *γδ* expression was similar to endogenous tissue.^[^ ^84^ ^]^ Analysis of T cells in the periphery revealed they too had normal gene expression of receptors. Unlike the ESCs methods previously described, here the authors created a model where they observed clear borders defining cortical and medullary regions of cTECs and mTECs. Regional differences were observed with the cortical areas expressing functional cTECs markers (*β*5T, CD205, DLL4) while mTECs expressed the tissue specific marker AIRE, which confirms a faithful resemblance of normal heterogeneous tissue. Despite not all grafts showed clear demarcation between cortical and medullary regions, this finding holds great importance. Studies have shown that about 25% of genes were differentially expressed between the cortex and medulla in healthy thymus tissue.^[^ ^85^ ^]^ A lack of thymic cell organization can cause the interaction between thymocytes and the stroma to skew VDJ recombination to favor the selection of certain TCRs, conceivably leading to a release of faulty naïve T cells into the periphery.^[^ ^81^ ^]^ Despite successfully reprogramming fibroblasts to iTECs, full reprogramming was not likely achieved, since endogenous markers like EpCAM, FOXN1, and TCR*β* were not highly expressed. It should also be noted that TECs were derived from murine embryonic fibroblasts, which might contain progenitor cells of unknown phenotype. Nevertheless, forcing the expression of FOXN1 is the most promising approach to date in creating organoids, which display regionalized gene expression and levels of T cell production.^[^ ^84^, ^86^ ^]^

Inspired by the promise in generating TECs through the forced expression of FOXN1 over‐expression, Otsuka et al. devised a protocol to develop TECs from iPSCs, through virally transducing FOXN1 expression.^[^ ^86^ ^]^ A high expression of TEC markers like Ly51^+^, UEA1^+^, DLL4, and MHCII was observed. Also, a tenfold higher amount of T cells in organoids vs controls occurred in vitro. However, after transplantation, the fibroblast induced TECs were only able to generate a small amount of T cells. This could be due to the levels of T cell chemoattractant CCL25 not being sufficient to allow T cell infiltration into the organoids. This could also possibly be due to the short survival time of TECs in vivo. Compared to previous methods, which generated TECs from PSCs, this study showed much more efficient TEC differentiation and strongly suggests FOXN1 promotes a TEC phenotype. However, the differentiation efficiency of TECs could be improved as only 10% of TEC expressed the marker EpCAM.

The transcription factor FOXN1 has been investigated further as a potential target to augment thymus function. During ageing the expression of FOXN1 is downregulated and is associated to thymic involution and diminished cTEC functionality.^[^ ^87^, ^88^ ^]^ Transgenic FOXN1 mice have shown an improved thymic architecture and resembled a juvenile thymus in terms of T cell output and gene expression profile.^[^ ^89^ ^]^ Furthermore, the importance of the transcription factor FOXN1 in the thymus was highlighted by its absence resulting in the sequestered expression of chemoattractants CCL25 and CXCL12, essential for T cell migration.^[^ ^90^ ^]^ Other studies have also supported the notion that increased levels of FOXN1 leads to increased thymic output and confer resistance to thymic involution^[^ ^91^, ^92^ ^]^

## 6 Organoids as Models of the Thymus

Organoids are defined as “an in vitro 3D cellular cluster derived exclusively from primary tissue or stem cells, capable of self‐renewal and self‐organization and exhibiting similar organ functionality.”^[^ ^93^ ^]^ Most organoid cultures do not have a niche that helps their growth in vivo. Therefore, artificial extracellular matrixes (ECM) are required to resemble native tissue. Past work has generated artificial skins and cartilage. However, these are relatively simple in comparison to the thymus heterogeneous tissue including its complex vasculature.

### 6.1 Adult Stem Cell Derived Thymus Organoids

Adult derived stem cells (AdSCs) can be differentiated into epithelial monolayers of the desired organ in the presence of correct niche factors. These epithelial monolayers contain distinct cells types and mimic the 3D nature of the organ created. Organoids including the liver, pancreas, lungs, and intestine have been grown in vitro.^[^ ^94^, ^95^, ^96^, ^97^ ^]^ As previously described, bipotent progenitor cells giving rise to both cTECs and mTECs have been observed and are presumed to reside in the cortical epithelial compartment.^[^ ^98^, ^99^ ^]^ Recent studies have uncovered possible TEC specific progenitor cells and have revealed their therapeutic potential in regenerating the thymic microenvironment.^[^ ^38^, ^100^ ^]^ For instance, Wong et al. isolated and identified thymic epithelial progenitor cells (TEPCs), which generated mTECs and cTECs in vitro in a stepwise fashion, which were able to multiply and self‐renew. TEPCs were characterized by low expression levels of MHCII and Ly51 and did not express the mTEC marker UEA‐1. Upon purification of TEPCs and engraftment into thymus nude mice, the cells retained their capacity to differentiate and could contribute to forming a cTEC and mTEC network.^[^ ^37^ ^]^ Ulyanchenko identified a different TEC subset PLET1(+)Ly‐51(+) present at the cortical medullary junction which was also able to generate both cTECs and mTECs for 9 months. Notably, these cells expressed EpCAM+FOXN1, markers associated with a TEC phenotype. Sekai et al. also found that embryonic mTEC stem cells, which expressed claudin‐3 and claudin‐4 (Cld3,4), could exclusively regenerate into mTECs following isolation and expansion in vitro. When transplanted, cells allowed the development and lifelong self‐tolerance of T cells.^[^ ^101^ ^]^

It remains to be seen whether the progenitor cells identified by Wong and Ulyanchenko et al. can be expanded on a clonal level. The addition of the correct niche factors to these progenitors cells and the knowledge of optimal culture conditions is needed to make organoids from a single stem cell.^[^ ^102^ ^]^ Nevertheless, characterization of these adult bipotent cells is important as they hold the potential to be targeted to improve thymus cellularity in disease.

#### 6.1.1 Thymus Cell Cultures Derived from Foetal and Adult Tissue

In addition to the expansion of bipotent TECs, isolation and expansion of normal thymic cells can yield normal phenotypic, physiologic, and functional properties. For instance, Villegas et al. created an enzyme free procedure where non fetal mTECs were isolated and expanded into thymic tissue. Expression of key molecules involved in processes such as immune tolerance, and the ability to respond to environmental cues such as inflammatory cytokines was maintained.^[^ ^103^ ^]^ However, these human derived thymus cultures are limited by the donor and fail to expand beyond 7–8 days.

Various groups have attempted to create cultures capable of inducing T cell development and differentiation from mouse foetal/post‐natal thymic tissue. The earliest assays used healthy foetal and adult thymic tissue to generate T cells and have been extensively described.^[^ ^104^ ^]^ Using a method called reaggregate foetal thymus organ culture (FTOC), researchers have been able to create a miniature thymus comprising of both cTECs and mTECs. Here, thymic cells are normally isolated from NOD SCID mice or post‐natal tissue. Cells are subsequently dissociated with deoxyguanosine and cultured on gel foam sponges. Thymic cells form aggregates that can be co‐cultured with HSC, which support T cell development following transplantation into athymic mice.^[^ ^105^, ^106^ ^]^ Thymic tissue showed great capacity for self‐organization and similarities in regional gene expression, while also able to resemble mechanisms to endogenous thymuses.

However, isolating fetal tissue samples can be challenging, and its bulky nature can decrease interactions between developing thymocytes, resulting in poor T cell development.

To circumvent this, the isolated tissue can be put into single suspensions and cultured on gelatin (Gelfoam) sponges in an air–liquid interface, which is termed reaggregate thymus organ culture (RTOC), held in a 3D conformation essential for T cell functionality. These assays can support T cell differentiation in vitro when HSCs are added to these aggregates. In addition, T cell differentiation is also supported in vivo when aggregates are transplanted into athymic mice.

FTOC/RTOC are good systems to study T cell development as various genetic modifications can be made through siRNAs, antibodies, and cytokines. These can have applications like improving processes such as positive selection by inducing the expression of MHC molecules. This method can also be used to investigate cells by flow cytometry and study gene expression through RT‐PCR and microarrays. However, high throughput of mTECs and cTECs through FTOC/RTOC has not yet been achieved and the resulting structures do not fully resemble a normal thymus. In addition, these systems lack efficiency and the ability to fully mediate positive and negative selection processes.^[^ ^106^, ^107^ ^]^

### 6.2 Biomaterials in the Presentation of Development Signaling Molecules in Thymus Modeling

The FTOC systems can be difficult to operate and cannot give quantitative information on human T cell development. In 2002, Schmid et al. developed a murine cell line, which could effectively differentiate into T cells. As previously described, Notch signaling is critical for T cell lineage commitment and development. They used the murine cell line 0P9 (expressed MHC1) derived from the bone marrow, virally transduced them to express DLL1, and co‐cultured cells in the presence of IL‐7 and other cytokines, which were termed OPDL1 (Figure 4b).^[^ ^108^ ^]^ This resulted in HSCs committing to the T cell lineage whereby functional CD8^+^ and *γδ* T cells were created at the expense of B cells. DLL1, which shares homology with DLL4, is likewise able to support T cell development in a similar assay.^[^ ^109^ ^]^

A limitation of this system is that these cells must be genetically modified, which can be cumbersome and interferes with the normal genetic makeup of OP9 cells. In addition, the use of this system requires high amounts of animal serum and is not very efficient in generating T cells.^[^ ^109^ ^]^ Positive and negative selection processes are also hampered, which could be in part due to OP9 cells failing to express key molecules such as MHCII, CD1d, and possibly AIRE, rendering them unable to positively select CD4^+^ T cells. Moreover, T cells do not show high expression of the CD3 TCR complex, which could lead to poor selection processes. Despite generated T cells showing responsiveness to stimulation, its efficacy does not match a normal thymus.^[^ ^93^, ^94^, ^95^ ^]^

Using the OPDL1 system does not allow to fine tune the density and orientation of the ligand DLL1 or DLL4 and to quantitate their effects. Potential solutions such as anchoring DLL4 to magnetic beads through strepavidin biotin and antibody binding has enabled the modulation of density and orientation. This system yielded high amounts of Thy1.2+ T cells, which suggests TECs HSCs contacts are not necessary to induce T cell development. Moreover, this preserved the ligand, which resulted in efficient Notch signaling and yielded a purer population of Thy 1.2^+^ cells (59.7%) compared to controls (33.4%), which did not have the Notch ligand present. However, the authors observed some B cell production indicating still some degree of inefficiency of the Notch bead culture system.^[^ ^110^, ^111^ ^]^ The OPDL1 co‐culture system lacks the complex thymic microenvironment, which provides cues for various processes. Consequently, attempts to make a more complete niche for T cell development have been modeled by immobilizing DLL4 to an alginate PEG cryogel scaffold capable of releasing BMP2, which could recruit bone marrow stromal cells.^[^ ^112^ ^]^

Despite the OPDL1 system showed to be competent in generating T cells, it lacks scalability. Therefore, strategies to enhance T cell output have been explored. The addition of the cell adhesion molecule VCAM‐1 to OP9 cell cultures has shown to synergistically act with DLL4 to enhance the recruitment of T cell precursors. The authors claimed this system could generate 1 × 10^7^ CD7^+^ (early surface marker in T cell ontogeny) progenitor T cells, a similar dose given to patients for allogeneic T cell immunotherapy.^[^ ^113^ ^]^ Cells also sequentially showed normal development. In vivo analysis revealed that T cells were able to respond to stimulation and produced IFN‐*γ* and IL‐2, at similar levels to controls. Other molecules like Wnt3a have been proposed, which could also aid large scale T cell production. However, a range of T cell functions like induction of tolerance, as well as activation of other immune cells, was not tested.^[^ ^114^ ^]^

#### 6.2.1 Artificial Organoid Systems

The OPDL1 system can show suboptimal negative and positive selection. For instance, a skew toward the production of CD8^+^ T cells is observed as well as T cell showing TCR abnormalities. In addition, Notch signaling, which is essential for T cell development, has shown to be disrupted in 2D cultures as it led to the loss of DLL1 and DLL4.^[^ ^115^ ^]^ Therefore, having a 3D architecture is imperative to mimic the environment of the thymus. Accordingly, novel 3D cultures have made use of aggregating HSCs together with MS5‐hDLL1 cells, a genetically modified murine stromal cell line expressing human DLL1 or DLL4. Seet et al. created an artificial thymic organoid (ATO) system whereby co‐cultured aggregates of HSCs and MS5‐hDLL1 cells were added on a cell culture insert at the air–fluid interface in a serum free medium (Figure 4c). This robustly supported T cell development from various sources of progenitor cells including BM, chord blood, and PB.^[^ ^93^ ^]^ T cell population rapidly increased and accounted for 30% of total generated cells at 6 weeks, which are levels comparable to a native thymus. Using the modified MS‐5 system yielded a superior amount of DP T cells with 78.5% efficacy, compared to the conventional OPDL1 system, which yielded 9.73% efficacy. In addition, positive selection was greater with organoids producing 74% SP CD3 TCR *αβ* T cells, with the conventional OPDL1 system yielding only 26% SP CD3 TCR *αβ* T cells. T cells showed similar development to normal T cells with a transition from an immune naive to a mature naive phenotype. Different subsets such as Th1, Th2, and Th17 were present. T cells had a diverse TCR gene and were able to produce cytokines in response to stimuli. For instance, RAG1 and RAG2, genes important for VDJ recombination, were expressed. Deep sequencing revealed similar usage of TCR*β* to naïve T cells from endogenous tissue. CD8^+^ T cells were responsive to PMA and ionomycin stimulation, as they produced IFN‐*γ* and TNF‐*α*. CD25 and 4‐1BB, markers associated with potent T cell activation and cytokine production, were also upregulated. The main limitation of this study was that a higher CD8^+^ to CD4^+^ T cell ratio (indicative of an impaired immune system) was found, opposite of what is normally observed.^[^ ^117^ ^]^ Long term survival of T cells was poor with a decrease of CD8^+^ T cells over time. However, organoids were relatively easy to set up and were intact for up to 20 weeks.

The ATO system has been applied in aiding the production of conventional T cells from ESCs and iPSCs. In short, embryonic mesodermal progenitors were created from ESCs or iPSCs and co cultured with MS5‐DLL4 cells in a liquid–air interface. Various compounds were added to induce T cell lineage (both CD4 and CD8) commitment, which showed responsiveness to stimulation, albeit at lower levels compared to a normal thymus.^[^ ^118^, ^119^ ^]^ Moreover, these ATO systems showed to form T cells with a similar transcriptional profile in isolated CD4 and CD8 compared to a normal thymus and peripheral blood.^[^ ^120^ ^]^

However, these systems make use of murine cell lines. The xenogeneic nature of the feeders, as well as of the murine cells themselves, would be problematic for their use in the clinic. Additionally, the long‐term generation of thymic cells can lead to a loss of aggregation capability for reasons which are still unknown. For example, Bonfanti et al. described organoids that could be cultured for up to 56 days. However, upon serial passaging, they lost their colony‐forming capacity and expression of TEC functional markers like MHCII and AIRE.^[^ ^121^ ^]^ In addition, outcomes in results can be heterogeneous between individuals and differ from group to group. Future studies need to further investigate the phenotype of T cell markers to indicate to what extent these systems support full T cell development.

Preliminary results of artificial organoid based systems and biomaterial‐based models of thymuses are promising. Protocols should be optimized to allow transplantation in humans. Bredenkamp et al. suggested that new assays should be developed to indicate the capacity of organoids to generate new T cells.^[^ ^70^ ^]^

## 7 Scaffolds as Aids in Thymus Regeneration

Thymic tissue created solely from cell‐based approaches or by reprogramming cells are unlikely to reach a scale feasible for transplantation. Production of thymus constructs on a large scale will most likely require scaffold structures, which would result in a sufficient output of T cells (Figure 4a).^[^ ^122^ ^]^ Scaffolds are synthetic or biological matrices, which can provide a structural frame on which cells or organoids can be grown, as a substitute for the natural ECM. In addition, matrices have been developed from decellularized tissues, which can provide a suitable environment for TECs repopulation. These constructs provide mechanical structural support for cells to propagate in. They also give biochemical signals, which regulate cell behavior, such as proliferation, differentiation, and migration. The choice of material used for scaffold construct yields great influence on tissue survival, therefore careful consideration should be taken.

### 7.1 Natural Polymers as Scaffolds for Thymus Regeneration

Natural polymers have been explored in thymus construction as support systems, since they have a high‐water content and are porous, which allows easy access for nutrients and facilitate proliferation. The ECM of thymus tissue is composed of high amounts of natural proteins in a state of “dynamic reciprocity” with TECs. It was reported that the most abundant ECM molecules in thymic tissue include collagen I, IV, as well as laminin and fibronectin.^[^ ^123^, ^124^ ^]^ Many studies have encapsulated TECs into these materials in the endeavor of creating TEC capable of T cell production. Bortolomai et al. developed a method where they grew TECs on 3D collagen type I scaffolds cross‐linked with other biological materials. They also virally induced TECs to transiently express Oct4 that increased TEC expansion, which faithfully mimicked the thymic microenvironment.^[^ ^125^ ^]^ The outcomes demonstrated that TECs had correct gene expression of thymic markers, such as FOXN1, DLL1, DLL4, and AIRE. The organoids were highly vascularized, demonstrated by neovascularization as early as 2 weeks post transplantation. This study highlights the possibility of gene modification in conjunction with scaffolds to create models of postnatal thymic regeneration. However, this system did not support thymopoiesis and T cells were short lived, similarly found in various other studies.^[^ ^125^ ^]^

3D scaffolds have also been applied to iPSC derived TECs unlike previous studies, which used 2D systems.^[^ ^67^, ^71^ ^]^ Okabe et al. added murine iPSC derived TECs to low attachment 96 U‐bottom well plates. This mediated the development of TECs embedded into their natural extracellular matrix (ECM), which yielded a similar expression of FOXN1 compared to controls and produced functional T cells. This 3D spheroid system could enable optimal spatiotemporal cell–cell interactions, thereby better mimicking the embryological development of the TECs. Moreover, isolated thymocytes showed tolerance to both donor and recipients MHC molecules, but were present in small quantities in secondary lymphoid organs.^[^ ^126^ ^]^ However, studies have indicated natural scaffolds do not fully support thymopoiesis and T cells were short lived. In addition, scaffolds had a short lifetime, indicated by phagocyte infiltration. To overcome potential degradation, the encapsulation of thymic constructs in gelatin‐based microgel solutions have been explored. This would potentially allow sturdy protection against external forces and would make it compatible for large scale bioreactor based expansion.^[^ ^127^ ^]^

In addition, a potential solution to the lack of T cells production by conventional scaffold systems was explored by Stachowiak et al. who encased fibrillar collagen matrices in a poly (ethylene glycol) (PEG) inverse opal hydrogel. The combined ability of collagen to mediate migration of T cells, with the robustness of the hydrogel, resulted in efficient T cell migration within the scaffold. In addition, modifications were made to influence the local microenvironment. The chemokine CCL21 was coupled to the hydrogels, which further supported lymphocyte migration. Hydrogels were able to facilitate 3D TEC aggregation and showed that TECs maintained their molecular and functional properties.^[^ ^128^ ^]^

Experiments using 2D cell cultures are not sufficient to represent physiological conditions seen in 3D, which is essential for TEC development. TECs cultured on 2D surfaces have shown to lose expression of key markers, such as CXCL12, as well as de‐differentiated into other cell types. In addition, TECs can undergo apoptosis. To overcome this, Pinto et al. constructed a 3D scaffold capable of mimicking the developmental biology of the mTECs.^[^ ^129^ ^]^ Briefly, dermal fibroblasts isolated from human skin were embedded into a fibrin gel. This was supported by a scaffold made from nonwoven fibrous material inserted into a polyester capillary pore membrane. mTECs were subsequently added and were able to rapidly proliferate from expressing markers CD80^lo^ and AIRE^−^ to CD80^hi^ and AIRE^+^, key markers of terminally differentiated cells. The TECs were capable of expressing multiple self‐peptides for both positive and negative selection processes, which were AIRE dependent or independent, termed promiscuous gene expression. Albeit not tested, this regionalized gene expression is critical to mediate negative selection. Furthermore, mTECs were also able to respond to cues such as RANKL, which allowed them to obtain AIRE expression.

The usage of natural materials for scaffolds to support organoids is desirable for their biodegradability, biocompatibility, bio‐absorbability, and low immunogenicity. Mammalian derived ECM proteins have natural ligands and signaling molecules, which means no further modifications need to be made for cell support. In addition, they are easily accessible from animals and closely resemble endogenous tissue.^[^ ^130^ ^]^ The use of 3D scaffold structures is imperative since they allow developing thymocytes to interact with selective ligands, that is, MHC, presented on TECs, otherwise not feasible in 2D culture systems. Mechanical properties of polymers can be controlled by adjusting the prepolymer density. Future studies should continue to address whether T cells develop and display functionality, by evaluating TCR rearrangement, cytokine production, and their ability to differentiate into other T cell subsets.

#### 7.1.1 Decellularized ECM Scaffolds for Thymus Regeneration

Although natural polymers display competence in providing a microenvironmental niche for TECs, the high diversity of naturally occurring ECM makes it challenging to duplicate. A commonly used technique for scaffold fabrication is decellularization. Here, the cellular constituents of an organ are cleared. However, the native structure and ECM components are preserved, which provides structural support and diverse molecular cues in the form of cytokines/chemokines and growth factors (Figure 4a). Several methods including physical, chemical, and biological methods are used to create decellularized tissues depending on factors such as lipid content and tissue thickness and density.^[^ ^131^ ^]^ The choice of the decellularization method influences the final properties of the obtained ECM. For instance, physical techniques can cause damage to the matrix, while chemical techniques can confer chemical changes to the ECM.^[^ ^132^ ^]^

Previous methods have successfully created ECM scaffolds from organs like the heart, gastrointestinal tract, liver, lungs, and kidneys, which were repopulated with parenchymal cells and have shown the potential of using human organoids in a clinical setting.^[^ ^102^, ^133^, ^134^ ^]^ In this regard, Fan et al. made use of a freeze–thaw decellularization method to create a natural 3D scaffold, where the ECM architecture (fibrillary network, grooves, and ridges) was preserved. Components of the thymic microenvironment including TECs, thymic fibroblasts, and endothelial cells were added to create thymus organoids. This structure was able to home lymphocytes and support thymopoiesis.^[^ ^81^ ^]^ An increased amount of CD3^+^ CD4^+^ CD8^+^ T cells in primary and secondary lymphoid organs was observed. In addition, the ratio of CD4^+^ to CD8^+^ T cells was similar to controls. Moreover, subsets of T cell like Tregs were produced at similar levels to endogenous thymic tissue. The thymic architecture expressed important T cell proliferation factors like Trp63 and Tbata.^[^ ^135^ ^]^ The rapid proliferation of cells was shown by expression of Ki67^+^ and EpCAM^+^. T cells displayed functionality as they rejected implanted foreign skins allograft in generally the same time (2–3 weeks) as controls. In addition, CD4^+^ T cells showed they could activate effector cells. Humoral immunity was established, since T cells were shown to be able to mediate affinity maturation and class switching in B cells. Following ovalbumin (OVA) stimulation, high levels of anti‐OVA antibodies (IgG2b and IgG3) were detected at similar levels to mice thymuses 4 weeks post‐immunization. Finally, CD8^+^T cells showed antigen specificity in response to a specific OVA peptide.

Despite this, the generation of T cells needs to be optimized, as they found that only about 10% of DP and SP thymocytes expressed the CD3 complex. In addition, T cells did not show rapid proliferation in response to stimulation compared to controls. The numbers of T cells in organoids were only 10% of T cells in naive mice in the periphery. Long term survival of TECs could be improved as they only sustained their specific molecular properties for up to 8 weeks.^[^ ^81^ ^]^ Using freeze–thaw decellularization was not able to support *ex vivo* thymopoiesis and is unlikely feasible for non‐murine thymi due to the much larger size.

Perfusion based decellularized techniques to create ECM for in vitro thymus cultures have also shown promise. Recently, Campinoti et al. performed whole organ perfusion of rat thymi followed by chemical and enzymatic solutions to create decellularized thymic lobes. Long‐term expanding human TECs were also identified and subsequently embedded in the scaffold. The perseveration of the 3D ECM network of the scaffolds allowed TEC growth. Furthermore, TECs were able to yield Hassall's bodies, normally unseen in other in vitro thymus cultures. T cells showed a CD4:CD8 ratio, similar to normal physiological conditions and were functional, indicating an instructive thymus stroma both in vitro and in vivo.^[^ ^136^ ^]^ Whole organ perfusion‐based models of other organs have shown to preserve ECM components and facilitate large scale production, conceivable too with TECs.

Decellularization agents used in organ decellularization such as acids, bases, and alcohol could result in some disruption of the ECM, which could have an effect on recellularization with TECs. Therefore, the selection of agents causing the least damage is vital. Hun et al. used cholamidopropyl dimethylammonium‐2‐hydroxy‐1‐propanesulfonate (CHAPS0), which productively cleared thymic components and retained critical ECM proteins. The architecture of TECs was preserved and was able to home HSCs. TECs matured and expressed markers like AIRE, and T cells were displayed functionality.^[^ ^137^ ^]^ This method of decellularizing tissue was shown to have several advantages over the RTOC method. For example, a peripheral T cell output count was much higher than the RTOC method, as well as higher levels of T cell activation and the number of memory CD4^+^T cells present.^[^ ^138^ ^]^ In contrast, other decellularization agents such as SDS has been associated with the loss of fibronectin, laminin, and collagen IV, critical for TEC survival. Other decellularization agents in this study, such as *n*‐octyl glucoside (NOG), were not completely effective in the decellularization process. Until recent advancements, the use of decellularized ECMs has been somewhat ineffective since thymic architecture does not fully resemble a normal thymus, which could explain why T cells were not fully functional and the immune cell population was relatively low in these studies.^[^ ^81^, ^137^ ^]^ Recently, Asnaghi et al. created decellularized thymic scaffolds from 6–8‐week‐old mice in a perfusion bioreactor. The procedure involved using hypotonic stress to mediate cell lysis as well as the addition of compounds for the removal of cellular debris. Importantly, major components of the ECM were preserved, which allowed TEC cultures to be sustained in vitro for over 30 days, essential to mediate long term thymopeosis. Notably, these constructs were also able to facilitate T cell development in vitro. In addition, upon engraftment into kidneys of athymic mice, constructs were also able to home HSCs. However, protocols could still be optimized, since the repopulation of scaffolds with recipient thymocytes was not always successful.^[^ ^139^ ^]^

Decellularization still needs to be optimized to clear the cellular components of the thymus, yet retaining proteins needed for TEC development. Fan et al. suggested a “top‐down approach” (breaking up and reassembling), which leads to the lack of demarcation between cortical and medullary regions, which can have a profound effect on processes such as VDJ recombination.^[^ ^140^ ^]^ A better understanding of the thymus ECM and their interactions with the thymic microenvironment would allow more accurate reproduction of cues that the endogenous tissue provide.^[^ ^141^, ^142^ ^]^

Recent advancements have been made to create decellularized scaffolds that could help improve recapitulating organ composition. Scaffolds created from a combination of decellularization, freeze‐drying, milling, gamma irradiation and neutralization resulted in a robust ECM hydrogel capable of supporting the growth of various endoderm derived organs. The fabricated scaffolds were clinically compatible and had a proteomic signature similar to the natural ECM.^[^ ^143^ ^]^ This suggests that dECM derived from organs with similar endodermal origins could support tissue development in vitro and could be applied to the thymus. A summary of the major decellularization techniques used in constructing thymus organoids is described in **Table** **1**.

**Table 1:** Summary of main aspects for decellularization of the thymus

Decellularization technique	Comments	Major findings	Reference
Freeze thaw and detergent	– ECM structure can be compromised during freezing. – Challenging to scale for human use.	– Support TEC growth in vitro. – T cells display wide range of functioning ability. – Long term survival lacking.	Fan et al. 2015 Tajima et al. 2015
Detergent based (CHAPSO) to create decellularized tissue	– Less damage than commonly used detergents and chemicals. – Challenging to scale for human use. – Possibility to denaturalize proteins.	– Supports TEC maturation – Higher T cell output compared to conventional RTOC method	Hun et al. 2017
Detergent/enzymatic based whole organ perfusion decellularization	– Provides niche in the form of ECM, protein support, and possibility of vascularization. – Preexisting damage and disease of tissue may affect success of decellularization.	– Various thymus cell types of present including HCs. – Possibility clinically applicable – Supports T cell development in vitro and in vivo.	Campinoti et al. 2020
Bioreactor based decellularization	– Higher degree of control of organ in size and shape. – Enhanced removal of cellular debris. – High physical force can cause damage.	– Sustained TEC survival in vitro (30+ days). – Facilitates T cell development in vivo and in vitro. – Repopulation of grafts still not optimal in vivo.	Asnaghi et al. 2021

### 7.2 Synthetic Materials as Scaffolds for Thymus Regeneration

Despite showing promise, dECM tissues need to be obtained from donors, which may hamper their use due to donor‐to‐donor variations and have limited availability. Alternatives include synthetic materials, which have also been used for scaffolding to generate TECs. They are not limited by donor's variability and can be fined tuned to obtain a more controlled microenvironment. For instance, Seach et al. added embryonic thymic tissue into silicone tubes with Matrigel and implanted them beside exposed epigastric vessels in athymic mice. This was a promising way to increase the amount of vascularization of the thymus.^[^ ^144^ ^]^ The authors observed that this system supported the growth of embryonic tissue when added to the silicone tubes. *αβ* CD4^+^ and CD8^+^ T cells increased after 2 weeks post implantation and were comparable to WT models. The growth of the implants was enhanced by FGF2 and sustained for up to 11 weeks. In addition, they were functional as they rejected mismatched skin grafts. Matrigel showed to an extent to support the formation of a thymus analogue capable of supporting T cell development. The numerous proteins and growth factors in Matrigel permit various cells to have a polarized morphology and mimic physiological behaviors. However, its derivation from a murine sarcoma line makes it inappropriate for clinical uses.^[^ ^145^ ^]^

Studies have made use of tantalum coated carbon scaffolds to encapsulate TECs and co‐cultured with HSCs, which allowed the development of functional T cells.^[^ ^146^ ^]^ However, recovered grafts were often fragmenting and had limited growth. Only one adult graft showed progression of naive to mature T cell development and it remains to be seen whether there is a broad repertoire of T cell functionality using these systems.

TECs can be sparse especially with increasing age. Alternatives such as the usage of non thymic epithelial cells and keratinocytes seeded on 3D tantalum carbon‐coated scaffolds have shown to support the development of T cells. These cells displayed key thymic markers such as FOXN1 and AIRE, albeit at a lower level. T cells developed normally from DP to SN, and by day 21 single positive CD4^+^ and CD8^+^ T cells were observed. Following concanavalin stimulation, T cells showed high levels of the activation marker CD60, suggesting they were functional.^[^ ^147^ ^]^ Despite mediating positive and negative selection in vitro, this system is very inefficient, and its reproducibility has been challenged.^[^ ^148^ ^]^ Methods to scale up the generation of T cells will need to be addressed if it is to be translated to the clinic.

Synthetic stiff material scaffolds, such as tantalum, have properties unsuitable for transplantation due to the thymus's inherent soft nature. In addition, the use of silicone as scaffolds has issues as it is not degradable. Large scale production remains challenging, and a mismatch of physicochemical properties is still present compared to the thymus. Each approach in constructing thymus organoids is summarized in **Table** **2**.

**Table 2:** Summary of main aspects for each regenerative approaches of the thymus and their advantages and disadvantages

Approaches	Comments	Advantages	Disadvantages	Notable references
1) OPDLL1system co‐cultured with HSCs	Can add synthetic DLL1 ligands and use of magnetic based DLL1 presentation for T cell differentiation. Enhance T cell differentiation by adding VCAM‐1 ligands.	Genetically modifiable to enhance T cell development and possible to study effects of various compounds. Efficient generation of T cells. T cells diverse, functional, and produce cytokines.	Genetic modification interferes with normal makeup and can be difficult. Hampered selection processes and poor T cell count and function. Cells fail to express key markers involved in selection processes.	Schmit et al., 2002 Awong et al., 2008 Taqvi et al., 2006
2) Differentiation of ESCs to TECs	Different protocols to induce TEC phenotype from ESCs.	Not limited by donors. Diverse T cell repertoire. Responsive T cells to stimulation.	Poor T cell count and fails to match endogenous T cell population in terms of functionality and diversity. Possibility of recipient rejection.	Parent et al., 2013 Sun et al., 2013 Su et al., 2015
3) Reprogramming cells to TECs	Simple protocol in generating TECs by a single transcription factor FOXN1. Till date most promising resemblance to endogenous tissue and T cell count.	Faithful resemblance of heterogenous tissue. Similar CD4:CD8 ratio to endogenous tissue.	In vivo poor survival. Scalability remains an issue. Unknown if feasible for different sources of blood.	Bredenkamp et al., 2014b Otsuka et al., 2020
4) Scaffolding for TEC generation	Decellularized tissue. Natural polymers. Synthetic polymers.	Large scale production possible. Decellularized tissue has natural components of TEC architecture. Polymers modifiable for desired properties.	Limited by number of donors. Some material unsuitable for human transplantation. Lacks endogenous architecture and T cell number & function.	Fan et al., 2015 Bortolomai et al., 2019 Seach et al., 2017
5) Artificial thymic organoids (ATOs)	MS5‐DLL1 cell line centrifuged and aggregated with HSCs. MS5‐DLL1 cells can be co‐cultured with ESC and iPSCs to produce T cells	3D architecture provides better cellular interactions. Proper T cell differentiation.	Long term expansion can be challenging. Skew toward CD8 T cell differentiation.	Seet et al., 2017 Montel‐Hagen et al. 2019 Vizcardo et al. 2013

## 8 Exogenous Regeneration Therapies

Various studies have investigated the regeneration of the thymus via *ex vivo* methods, to enhance thymic function or to better mediate the development of T cells (Figure 5). Interleukins, such as IL‐15, have shown to increase TEC numbers, while IL‐22 has shown to act on TECs to mediate repair, possibly by the regulation of FOXN1 expression. The positive effects of IL‐22 on thymus regeneration are limited to a damaged state, as IL‐22 treatment in healthy mice showed no increase in cellularity.^[^ ^149^ ^]^ IL‐7 has shown to promote thymus repair and is critical for thymocyte development, as deletion of IL‐7 in TECs profoundly reduced the number of T cells.^[^ ^150^ ^]^ Currently, IL‐7 is being used to improve lymphocyte levels in COVID‐19 patients.^[^ ^151^ ^]^ Finally, IL‐21 can enhance hematopoietic reconstitution upon treatment, while IL‐21 has shown to enhance thymic function in mice.^[^ ^152^ ^]^

Candidate molecules such as KGF, which promotes TEC proliferation and differentiation, have been used to boost thymus function after damage. TECs express receptors (FGFR2IIB), for KGF and upon KGF treatment organization of the cortex and medulla was restored in mice.^[^ ^3^, ^153^ ^]^ However, its efficacy in humans is questionable as oral mucositis patients receiving KGF failed to increase thymic function.^[^ ^154^ ^]^ Other biological factors, such as stem cell factor (SCF) and the FMS‐like tyrosine kinase 3 ligand (FLT3LG), act on the hematopoietic compartment to increase thymopoesis.^[^ ^155^ ^]^

Interestingly, therapies that do not directly target the thymus, such as adoptive T cell therapy, has shown to improve thymus function. Adoptive T cell therapy can improve thymic cellularity as well as graft vs tumor activity.^[^ ^156^, ^157^ ^]^

### 8.1 Modulation of Endogenous Repair Pathways

The modulation of signaling pathways that influence TEC repair has shown promise in thymic regeneration. Wertheimer et al. showed that endothelial cells play a role in the restoration of the thymus by increasing the production of BMP4, which increased the expression of FOXN1 and its downstream target DLL1 to induce the development of TECs. BMP4 is a critical component in thymus regeneration as the administration of Noggin, a potent BMP inhibitor, slowed down thymus recovery.^[^ ^158^ ^]^ Collectively, these findings indicate that therapies upregulating FOXN1 could be used for thymic regeneration. It remains to be seen whether these could be translated to non‐murine models.

Other factors which have implications in the endogenous repair of the thymus have also been explored. For instance, the RANK ligand (RANKL) expressed on ProT cells has a key role in TECs fate. It can interact with RANK expressed on TECs triggering TEC differentiation through the regulation of mTEC transcription factor AIRE. Its important role in regeneration was evident when high upregulation of RANKL in CD4 lymphocytes was observed after cytoreductive conditions. Moreover, mice that were given recombinant soluble RANKL showed potent regenerative capacity which included improved TEC cellularity and architecture, while transgenic RANKL mice have shown similar findings.^[^ ^159^ ^]^

#### 8.1.1 Sex Steroid Inhibition to Enhance Thymus Recovery

As previously described, an increase of sex steroids during puberty is associated with thymic involution. This has been described in young mice, which showed profound atrophy of the thymus when given testosterone.^[^ ^160^ ^]^ Sex steroid removal through surgical or chemical castration has indicated thymus recovery and can boost lymphocyte production likely through the increased expression of CCL25 and DLL4.^[^ ^162^, ^163^ ^]^ Androgen receptor blockers, such as gonadotropin‐releasing hormone agonist (GnRH), have also demonstrated an increase in thymic cellularity and recovery of naïve T cell production.^[^ ^163^ ^]^ In addition, luteinizing hormone receptor agonists (LHRH), such as Lupron, which leads to the inhibition of luteinizing hormone (LH) and follicle stimulating hormone (FSH), have also been investigated. Emerging evidence indicates they increase the levels of CD4 T cells likely through facilitating the transcription of DLL4, and have shown success in murine studies.^[^ ^164^, ^165^ ^]^

LHRH agonists are the gold standard for ablation of sex steroids in prostate cancer patients and after hematopoietic cell transplantation. Recently, LHRH antagonists have been used for cancer patients.^[^ ^162^ ^]^ In addition, Goserelin, another LHRH agonists, was given to a cohort of patients prior to stem cell transplantation. It was shown to significantly increase levels of naïve T cell as well as TCR diversity post stem cell transplantation.^[^ ^166^ ^]^ Despite showing promising results, the ablation of sex steroids will have systemic effects. A deeper understanding of the specific roles that sex steroids play in the role of thymus involution is needed, as well as their specific targets within the thymus.

Lepletier et al. described that follistatin may be causing the post pubertal deterioration of TECs. During ageing, hormones like follistatin increase, which competitively binds to Activin A, negating its function. The protein Activin A increases the synthesis of FSH in the pituitary gland while its counterpart Inhibin decreases it. These proteins have been shown to influence TEC homeostasis. Indeed, Activin A knockout in mice have shown a reduction in TECs and displayed poor architecture, while Inhibin knockout increased mTEC differentiation. Interestingly, in an in vitro model, administration of Activin A and BMP were found to interplay in TEPC homeostasis. Activin A induced TEPC differentiation while BMP4 supported progenitor maintenance. These findings suggest Activin A could be targeted to promote recovery in TECs.^[^ ^167^ ^]^

Neuroendocrine hormones, such as growth hormone, have been proposed to restore thymus activity. For instance, growth hormone and IGF have shown to increase thymocyte number and increase thymus mass in mice. Likewise, hormones such as leptin, ghrelin, and thymosin *α*1 have all been associated with increasing thymus cellularity and having protective effects on thymopoiesis.

## 9 Emerging Technologies in Thymus Regeneration

Exogenous therapies in thymus renewal are encouraging, though their use will invariably come with side effects. Exogenous therapies are simply not a solution for thymectomy patients or patients with very few existing TECs. Techniques like organoids and cell‐based approaches bypass the need for an endogenous thymus and have shown to produce T cells. Notwithstanding, they are still not scalable and are yet to fully mirror organ morphology and functionality. Uncovering the mechanisms of thymocyte‐TEC interaction could enable the development of scaffolds allowing optimal crosstalk, which would enable the creation of more precise models capable of recapitulating TEC physiology and producing higher amounts of T cells.^[^ ^168^ ^]^

Accordingly, Park et al. used single cell sequencing (scRNA) and TCR sequencing to identify and uncover the heterogeneity of human thymus cells. They characterized different cell states from embryogenesis to the adult phase of the thymus and specified the distinct roles, which thymic cells play in thymopoiesis. In addition, novel subpopulation of thymic fibroblasts and epithelial cells were identified, as well as specific roles that they have in aiding thymocyte development. This will be instrumental in the creation of future models to assess functional aspects of thymus organoids and better model T cell development.^[^ ^169^ ^]^

Emerging technologies such as biofabrication could be used to improve approaches of in vitro thymus models. Various 3D bioprinted tissues have been created and revealed functional activity both in vitro and in vivo. Several groups have used additive manufacturing (AM) to incorporate defined structures into tissue models. For instance, AM has been used to deposit endothelial cells to increase vascularization and could be applied to thymic regeneration.^[^ ^130^ ^]^ AM can also create tubular structures, which could promote vascularization in vitro and in vivo. Examples include using a carbohydrate glass as a template for the fabrication of a vascular network. After its removal, channels remained intact, which were able to diffuse oxygen and nutrients.^[^ ^170^ ^]^ In addition, a recent study applied a self‐assembled honeycomb nanofibrous scaffold to support angiogenesis. This scaffold had a large surface area with high permeability, which could be an asset for designing thymic blood micro‐vessels.^[^ ^171^ ^]^

Scaffold systems used in thymic construct show great promise as they provide robust structural support for TECs and act as a substitute for naturally occurring ECM. The ECM has shown to be able to mediate signaling pathways like RHO and ERK, needed for stem cell differentiation. Molecules like CRY61 and Laminin‐211 are present in the ECM, which influence TEC morphology and functionality.^[^ ^81^ ^]^

Bioprinting could be applied to form a robust instructive ECM, which would moderate the spatiotemporal communication between cells and cells to ECM. Bioprinting of natural molecules like collagen, elastin, keratin, which are essential components of the ECM, could help make ideal scaffolds for TEC generation. In addition, these proteins can be modulated (by concentration) to match ideal conditions for TECs to propagate in. Studies have shown the ability to bioprint collagen scaffolds in shapes like tubes, sheets, sponges, and patient specific 3D geometries. This, in particular, could be useful for thymic regeneration as collagen scaffolds have already been found to reproduce a similar microenvironment to the endogenous thymic properties.^[^ ^172^ ^]^

Bioinks, the cell‐laden hydrogel biomaterials that are typically used for bioprinting, can be composed of decellularized ECM (dECM). As dECM bioinks are often too soft to self‐sustain their own weight, they can be deposited by using other polymers as supports to increase their strength. For example, Skardal et al. made use of bioinks composed of liver dECM, which supported the growth of hepatocytes and Kupffer cells.^[^ ^173^ ^]^ Other approaches could include developing bioink formulations comprising of growth factors, collagens, glycosaminoglycans, and elastin, which could achieve a better strength of the final bioprinted construct. New bioprinting technologies can facilitate the simultaneous deposition of many compounds, which could be an asset considering the heterogeneity of the thymus. Various molecules can be added to the bioinks to modulate aspects, such as viscosity, resolution, stiffness, and degradability of the bioprinted constructs.^[^ ^174^ ^]^ Full replication of the ECM has not yet been realized, since its organization is spatially specific and the bioinks conformation can be disrupted during preparation.^[^ ^175^ ^]^ Bioprinting has also the potential to create large‐scale products, which currently remains a challenge for thymic tissue.^[^ ^176^ ^]^ With other integrated biofabrication techniques, it could be also possible to monitor TECs activity and maturation in real‐time, while providing information about physiological changes in the fabricated 3D tissue model construct. However, it remains challenging to bioprint natural materials and mimic endogenous properties for such soft tissues like glands.^[^ ^176^ ^]^

Bioassembly techniques have been recently described in generating TECs. Tajima et al. used self‐assembling molecules to promote aggregation of TECs, which created functional T cells. They used a self‐assembling hydrogel, an amphiphilic peptide, EAK16II coupled to its analogue EAKIIH6, which formed a sturdy complex of *β*‐sheet fibrils with a His‐tag attached to it. When an adaptor complex composed of a recombinant protein loaded with an anti‐His and anti‐EpCAM antibodies was used, it resulted in TECs forming 3D clusters. This system allowed to fine tune and control the microenvironment and yielded functional T cells 5 weeks post transplantation. However, this system was not able to create clear distinct regions between the cortical and medullary regions.^[^ ^177^ ^]^ A similar study made use of two self assembling hydrogels capable of recruiting IgGs in vivo. Through attaching EpCAM to this complex, TECs were recruited. TECs were able to home naïve immune cells and could mediate their differentiation into *γδ* T cells and T regs.^[^ ^178^ ^]^

The aforementioned systems, in particular, are useful to create specific depositions of cells in a 3D specific configuration. This would be able to optimize interactions between TECs and developing thymocytes in a spatiotemporal manner, paramount for TEC persistence and proliferation.^[^ ^179^, ^180^ ^]^

## 10 Conclusion

The thymus is a complex organ capable of generating a vast repertoire of T cells equipped to fight against various pathogens. It is a critical part of the immune system but is highly prone to insults, highlighting the importance of developing methods to abrogate it. Systemic therapies may have side effects and alternatives should be investigated further. Organoids have shown a lot of promise and the ability to mimic endogenous thymic activity. However, a functional thymic microenvironment with heterogeneous tissue resembling the structural and functional characterization of endogenous tissue has not yet been achieved. Scaffolds can provide structural and molecular support for organoids and have somewhat been successful in aiding large‐scale production and a more faithful resemblance to the thymus. New directions in the use of synthetic or non‐synthetic materials to precisely control structural and molecular support will be essential to develop thymic cells on a large scale. Emerging techniques like biofabrication will significantly contribute to making large scale models, as well as creating constructs more capable of mimicking the endogenous tissue of the thymus gland, in the endeavor to translate them to the clinic.

## Conflict of Interest

The authors declare no conflict of interest.

## Author Contributions

H.S. wrote the article and designed figures. L.M. wrote and edited the article and supervised. All the authors discussed and corrected the manuscript.
